# Associations of Human Milk Oligosaccharides and Bioactive Proteins with Infant Morbidity and Inflammation in Malawian Mother-Infant Dyads

**DOI:** 10.1093/cdn/nzab072

**Published:** 2021-04-29

**Authors:** Josh M Jorgensen, Rebecca Young, Per Ashorn, Ulla Ashorn, David Chaima, Jasmine C C Davis, Elisha Goonatilleke, Chiza Kumwenda, Carlito B Lebrilla, Kenneth Maleta, John Sadalaki, Sarah M Totten, Lauren D Wu, Angela M Zivkovic, Kathryn G Dewey

**Affiliations:** Department of Nutrition, University of California, Davis, Davis, CA, USA; Department of Nutrition, University of California, Davis, Davis, CA, USA; Center for Child Health Research, Faculty of Medicine and Health Technology, Tampere University, Tampere, Finland; Tampere University Hospital, Department of Pediatrics, Tampere, Finland; Center for Child Health Research, Faculty of Medicine and Health Technology, Tampere University, Tampere, Finland; Department of Community Health, University of Malawi College of Medicine, Blantyre, Malawi; Department of Chemistry, University of California, Davis, Davis, CA, USA; Department of Chemistry, University of California, Davis, Davis, CA, USA; Department of Community Health, University of Malawi College of Medicine, Blantyre, Malawi; Department of Food Science and Nutrition, School of Agricultural Sciences, University of Zambia, Lusaka, Zambia; Department of Chemistry, University of California, Davis, Davis, CA, USA; Department of Biochemistry and Molecular Medicine, University of California, Davis, Davis, CA, USA; Department of Community Health, University of Malawi College of Medicine, Blantyre, Malawi; Department of Community Health, University of Malawi College of Medicine, Blantyre, Malawi; Department of Chemistry, University of California, Davis, Davis, CA, USA; Department of Chemistry, University of California, Davis, Davis, CA, USA; Department of Nutrition, University of California, Davis, Davis, CA, USA; Foods for Health Institute, University of California, Davis, Davis, CA, USA; Department of Nutrition, University of California, Davis, Davis, CA, USA

**Keywords:** human milk oligosaccharides, bioactive breast milk proteins, infant morbidity, infant inflammation, fucosyltransferase 2, FUT2, secretor

## Abstract

**Background:**

Human milk oligosaccharides (HMOs) and bioactive proteins likely benefit infant health, but information on these relations is sparse.

**Objectives:**

We aimed to examine associations of milk content of HMOs and bioactive proteins with incidence and longitudinal prevalence of infant morbidity (any illness, fever, diarrhea, acute respiratory infection, and loss of appetite) and markers of inflammation [C-reactive protein (CRP) and α-1-acid glycoprotein (AGP)]. These are secondary analyses of a randomized controlled trial.

**Methods:**

Breast milk samples at 6 mo postpartum (*n*  = 659) were analyzed to quantify absolute abundance of HMOs, relative abundance of fucosylated HMOs, sialylated HMOs, and 51 individual HMOs, and concentrations of 6 bioactive proteins (lactalbumin, lactoferrin, lysozyme, antitrypsin, IgA, and osteopontin). We examined associations of these constituents with infant morbidity from 6 to 7 and 6 to 12 mo, and CRP and AGP at 6 and 18 mo, considering maternal secretor status [presence or absence of the functional enzyme encoded by the fucosyltransferase 2 gene (*FUT2*) ] and adjusting for covariates and multiple hypothesis testing.

**Results:**

In secretors there were positive associations between total HMOs and longitudinal prevalence of fever (*P* = 0.032), between fucosylated HMOs and incidence of diarrhea (*P* = 0.026), and between lactoferrin and elevated CRP at 18 mo (*P* = 0.011). In nonsecretors, there were inverse associations between lactoferrin and incidence of fever (*P*  = 0.007), between osteopontin and longitudinal prevalence of lost appetite (*P*  = 0.038), and between fucosylated HMOs and incidence of diarrhea (*P* = 0.025), lost appetite (*P* = 0.019), and concentrations of AGP and CRP at 6 mo (*P* = 0.001 and 0.010); and positive associations between total HMOs and incidence of lost appetite (*P* = 0.024) and elevated CRP at 18 mo (*P*  = 0.026), between lactalbumin and incidence of diarrhea (*P* = 0.006), and between lactoferrin and elevated CRP at 18 mo (*P* = 0.015).

**Conclusions:**

Certain HMOs and bioactive proteins were associated with infant morbidity and inflammation, particularly in nonsecretors. Further research is needed to elucidate the causality of these relations.

This trial was registered at clinicaltrials.gov as NCT01239693.

## Introduction

Human milk contains a plethora of beneficial constituents for the growing infant. In addition to key macro- and micronutrients, it contains bioactive components such as human milk oligosaccharides (HMOs) and certain milk proteins that protect the infant from pathogenic invasion in a number of ways (1, 2). These include provision of metabolic substrates to enhance the growth of beneficial intestinal bacteria (3–5), mimicking receptors on the intestinal lining to which pathogens bind (6–8), competitively binding intestinal receptors (9, 10), acting as bacteriostatic or bactericidal agents (11–13), and modulating the infant's immune response (1, 3).

More than one-third of deaths globally in children aged 1–12 mo in 2016 were caused by lower respiratory infections (22.7% of deaths; 338,000 deaths) and diarrheal diseases (15.5%; 231,000 deaths) (14). Although the infant mortality rate has decreased in recent years, there remains a need for additional interventions to improve child survival and health. In vitro, ex vivo, and animal model studies have shown that particular HMOs and bioactive proteins in human milk are associated with inhibition of pathogens linked with lower respiratory infection and diarrheal diseases (15, 16) and modulation of immune cells (17). A limited number of studies of infants have shown decreases in lower respiratory and gastrointestinal infections in those who consume a formula fortified with particular HMOs or bioactive proteins (17–19). There is also evidence that particular HMOs and bioactive human milk proteins are associated with infant growth and development (20–25). One mechanism by which HMOs and bioactive proteins can enhance growth and development is by decreasing the incidence and prevalence of infections and decreasing inflammation (21, 26).

Thus far, studies of associations between HMOs or bioactive proteins and infant morbidity or inflammation have focused on a small number of human milk constituents. We analyzed >50 individual HMOs and 6 milk bioactive proteins from participants in a randomized trial in semirural Malawi to test the hypothesis that greater relative abundances of bioactive proteins and HMOs would be associated with decreased infant morbidity and markers of inflammation. We considered associations with the 6 bioactive proteins and 3 groups of HMOs (absolute abundance of all HMOs, and relative abundance of fucosylated or sialylated HMOs) as primary analyses. Because the fucose and sialic acid decorations on certain HMOs act as decoys for intestinal binding or competitively inhibit pathogen binding to cell receptors, we expected to see stronger inverse associations with morbidity and inflammation outcomes for decorated HMOs than undecorated HMOs. We considered as exploratory analyses the associations of infant outcomes with 2 groups of HMOs—HMOs that are both fucosylated and sialylated, and those that are undecorated (nonfucosylated neutral)—as well as with individual HMOs.

## Methods

This study is a secondary analysis of a randomized, controlled, outcome assessor-blinded intervention trial of mother-infant dyads conducted in a partly semiurban, partly rural area of the Mangochi District in Malawi. The iLiNS Project DYAD-Malawi trial, including sample size calculations, has been described elsewhere (27). Briefly, women were enrolled during pregnancy at ≤20 gestational weeks and randomly assigned to 1 of 3 interventions: *1*) iron and folic acid during pregnancy and placebo (low-dose calcium) for 6 mo postpartum; *2*) multiple micronutrient supplements during pregnancy and for 6 mo postpartum; or *3*) lipid-based nutrient supplements (LNSs) during pregnancy and for 6 mo postpartum. Infants of women in the LNS group consumed an infant version of the LNS from 6 to 18 mo. All children were followed up to 18 mo of age. Participants signed informed consent before enrollment into the study and authorized all future uses of their data in published research. Identifying data were maintained by a statistician not involved in the study and were not shared with the authors. The study protocol was approved by the Institutional Review Board, University of California, Davis; the College of Medicine Research and Ethics Committee, University of Malawi; and the Ethics Committee of Pirkanmaa Hospital District, Finland.

At enrollment, trained study staff collected sociodemographic information including age, parity, education, and socioeconomic status (including household assets and food insecurity). The household asset *z*-score (HHAZ) includes information on building materials of the house, sources of water, type of lighting used in the house, type of cooking fuel used in the house, and sanitary facility. HHAZ and food security indices were created as previously described (28, 29). Additionally, maternal height and weight were measured (and used to calculate BMI) in triplicate using high-quality scales (SECA 874 flat scale; Seca GmbH & Co.) and stadiometers (Harpenden stadiometer; Holtain Limited). HIV status was assessed using a whole-blood antibody rapid test (Alere Determine HIV-1/2; Alere).

Breast milk was collected at 6 mo postpartum. Because of the variance in HMO content of breast milk over time (30, 31), we only included data from breast milk samples that were collected 2 wk before or after the planned collection date at 6 mo postpartum. Breast milk collection and analysis of HMOs and bioactive proteins were described previously (32). Briefly, mothers manually expressed the full content of 1 breast into a sterile plastic cup. Study staff thoroughly mixed the contents and then transferred 10 mL to storage cryovials, which were stored at −80°C until analysis. HMOs were analyzed by nano-LC-chip time-of-flight MS (33). We report absolute abundance of all the HMOs as ion counts, and we report the relative abundances of the groups of HMOs (fucosylated, sialylated, fucosylated and sialylated, and undecorated HMOs) and individual HMOs as proportion of all HMOs. We did not have a measurement of the quantity of breast milk consumed, so for the HMO analyses we deemed relative abundance as a better predictor of intake for these analyses than the absolute abundances. Lactoferrin, lactalbumin, lysozymes, antitrypsin, IgA, and osteopontin were analyzed by multiple reaction monitoring (34). All the proteins except for osteopontin are reported as grams per liter. Standards were not available for osteopontin at the time of analysis, so the results are reported as absolute abundance (ion counts).

At 6- and 18-mo planned study visits, clinic nurses collected 5 mL infant blood from the antecubital vein into a 7.5-mL trace mineral–free polypropylene syringe (Sarstedt Monovette, NH4-heparin; Sarstedt Inc.). The blood tube was immediately inverted 10 times to mix the heparin anticoagulant with the blood to prevent clotting. The tube was then placed in an insulated cooler with ice packs until processing. At the time of processing, trained laboratory staff centrifuged the whole blood at 1100 × *g* at room temperature for 15 min and separated plasma into storage cryovials. The storage vials were placed upright in freezer boxes in a −20°C freezer for temporary storage at the satellite clinics. Within 48 h, drivers transported the plasma to the main laboratory for long-term storage at −80°C.

Plasma was shipped on dry ice (World Courier) to the Western Human Nutrition Research Center at University of California, Davis for analysis. We analyzed C-reactive protein (CRP) and α-1-acid glycoprotein (AGP) from those samples by immunoturbidimetry on the Cobas Integra 400 system autoanalyzer (F. Hoffmann-La Roche Ltd). We analyzed all the samples in singlet, except for 5% of the samples, which we randomly selected to be analyzed in duplicate. None of those samples run in duplicate had a CV >5%. We defined high CRP as >5 mg/L and high AGP as >1 g/L.

Trained field workers visited participants’ homes every 7 d to deliver supplements and collect child morbidity information. Caregivers were asked about symptoms of illness in the previous 7 d using a structured questionnaire with pictures to aid with recall. Additionally, caregivers were provided with a picture calendar each week to prospectively collect information on their child's symptoms on a daily basis. These techniques were performed to minimize problems associated with recall in community morbidity assessments (35). Morbidity information from nonscheduled clinic and hospital visits made when the child was sick was also collected by trained study staff. In addition to receiving complimentary nutrition supplements, participants were compensated monetarily for time and travel expenses to clinical study visits. Participants were compensated with rice and/or soap for home visits.

Morbidity categories were created based on symptoms. Diarrhea was defined as ≥3 abnormally loose stools in a 24-h period. Acute respiratory infection (ARI) was defined as cough, rapid or difficult breathing, and nasal discharge. Fever was defined as caregiver's perception of high body temperature. Loss of appetite was defined as unwillingness to eat. “Any illness” was defined as presence of any of the above illnesses. We examined both short-term (6–7 mo) and long-term (6–12 mo) intervals when creating the morbidity variables for these analyses. During each interval, incidence of illness was defined as the number of new episodes of symptoms that followed ≥2 symptom-free days. The longitudinal prevalence of illness was the percentage of reported days of illness symptoms within the days morbidity data were available for each symptom.

The concentrations of the bioactive proteins and abundances of groups of HMOs examined as predictor variables are listed in **Table 1**, whereas the names and specific components of the individual HMOs are listed in **Table 2**. We prespecified as primary analyses the models for the associations of outcomes with all 6 proteins and 3 general categories of HMOs [absolute abundance of all HMOs (total HMOs), and relative abundance of fucosylated HMOs and sialylated HMOs]. We expected the bioactive proteins and groups of HMOs to be inversely associated with infant morbidity and markers of inflammation. We considered as exploratory analyses the associations of morbidity and inflammation with the relative abundance of 3 groups of HMOs (those with an α1-2-linked fucose, those that are both fucosylated and sialylated, and those that are undecorated—nonfucosylated neutral HMOs), as well as the individual HMOs detailed in Table 2.

**TABLE 1:** Concentrations of bioactive proteins and abundances of groups of HMOs in Malawian secretors (positive for the functional enzyme encoded by the *FUT2* gene) and nonsecretors^1^

Variable	Description	Concentration or abundance in secretors (*n* = 485)^2^	Concentration or abundance in non-secretors (*n* = 162)^2^
Antitrypsin	Protein, g/L	0.035 (0.026, 0.044)	0.034 (0.026, 0.044)
IgA	Protein, g/L	0.32 (0.25, 0.39)	0.33 (0.26, 0.43)
Lactalbumin^3^	Protein, g/L	1.30 (1.18, 1.50)	1.34 (1.19, 1.49)
Lactoferrin^4^	Protein, g/L	0.98 (0.75, 1.25)	0.95 (0.71, 1.16)
Lysozyme	Protein, g/L	0.05 (0.03, 0.07)	0.05 (0.03, 0.08)
Osteopontin^5^	Protein (ion counts)	73,555 (18,047,102,778)	69,377 (21,147,103,241)
Total HMO*	Absolute abundance of all HMOs (ion counts)	0.70 (0.58, 0.83)	0.57 (0.46, 0.67)
Fucosylated HMO*	Relative abundance of fucosylated HMOs, %	64 (59, 68)	57 (44, 63)
Sialylated HMO*	Relative abundance of sialylated HMOs, %	11 (9, 13)	15 (13, 17)
HMO with α1-2-linked fucose*	Relative abundance of HMOs with α1-2-linked fucose	21 (17, 26)	1.5 (1.0, 2.0)
Fucosylated and sialylated HMO*	Relative abundance of HMOs that are both fucosylated and sialylated, %	3.5 (2.2, 4.8)	4.8 (3.3, 6.8)
Undecorated HMO*	Relative abundance of nonfucosylated neutral (undecorated) HMOs, %	28 (24, 34)	34 (27, 45)

^1^The analyses among bioactive proteins and groups of total HMOs, fucosylated HMOs, and sialylated HMOs were considered primary analyses. Analyses among the groups of HMOs that were both fucosylated and sialylated and the nonfucosylated neutral (undecorated) HMOs were considered exploratory. *Median values differed between secretors and nonsecretors. CRP, C-reactive protein; *FUT2*, fucosyltransferase 2; HMO, human milk oligosaccharide.

^2^Values are median (25th, 75th percentile).

^3^There were interactions between lactalbumin and secretor status for longitudinal prevalence of diarrhea and incidence of any illness from 6 to 7 mo.

^4^There were interactions between lactoferrin and secretor status for longitudinal prevalence of lost appetite and incidence of fever and lost appetite from 6 to 12 mo, and elevated CRP at 18 mo.

^5^There were interactions between osteopontin and secretor status for incidence and longitudinal prevalence of lost appetite from 6 to 12 mo.

**TABLE 2:** Names, compositions, and relative abundance of the HMOs analyzed in Malawian women^1^

Abbreviation^2^	Composition^3^	Name	Relative abundance among secretors^4^ (*n* = 485)^5^	Relative abundance among nonsecretors (*n* = 162)^5^
3′SL*	2001	3′-Sialyllactose	2.1 (1.6, 2.9)	3.1 (2.1, 4.0)
6′SL*	2001	6′-Sialyllactose	0.09 (0.03, 0.16)	0.21 (0.09, 0.50)
3FL*	2010	3-Fucosyllactose	0.22 (0.09, 0.47)	0.60 (0.18, 1.07)
2′FL*	2010	2′-Fucosyllactose	14 (10, 18)	0.32 (0.18, 0.51)
LDFT*	2020	Lactodifucotetraose	4.2 (1.0, 8.1)	0.08 (0.05, 0.15)
LNT*	3100	Lacto-*N*-tetraose	14 (11, 17)	22 (16, 30)
LNnT*	3100	Lacto-*N*-neotetraose	8.5 (6.5, 10.5)	6.8 (3.9, 9.4)
LNT + LNnT*	3100	Lacto-*N*-tetraose + lacto-*N*-neotetraose	23 (19, 27)	29 (23, 37)
LSTa*	3101	Sialyllacto-*N*-neotetraose (a)	0.35 (0.22, 0.51)	0.45 (0.27, 0.73)
LSTb*	3101	Sialyllacto-*N*-tetraose (b)	2.0 (1.5, 2.4)	3.3 (2.7, 3.8)
LSTc*	3101	Siallylacto-*N*-tetraose (c)	1.7 (1.3, 2.2)	1.8 (1.3, 2.4)
LNFP I + III*	3110	Lacto-*N*-fucopentaose I + III	5.6 (3.7, 9.6)	3.6 (2.2, 4.4)
LNFP II*	3110	Lacto-*N*-fucopentaose II	4.1 (0.8, 6.1)	9.6 (0.9, 11.3)
F-LSTc*^6^	3111	Monofucosylmonosialyllacto-*N*-neotetraose	0.18 (0.09, 0.35)	0.15 (0.08, 0.56)
LNDFH + 3120*	3120	Lacto-*N*-difucohexaose I + II + unknown 3120	2.2 (0.3, 3.3)	3.4 (0.5, 4.3)
4100a	4100	No literature name	0.12 (0.08, 0.23)	0.15 (0.09, 0.26)
4100b*	4100	No literature name	0.09 (0.06, 0.12)	0.12 (0.09, 0.18)
LNH*^7^	4200	Lacto-*N*-hexaose	0.72 (0.39, 1.24)	0.65 (0.31, 1.9)
LNnH*	4200	Lacto-*N*-neohexaose	1.7 (1.0, 2.5)	1.1 (0.4, 2.0)
p-LNH	4200	*para*-Lacto-*N*-hexaose	0.38 (0.19, 0.81)	0.23 (0.10, 0.45)
S-LNH*	4201	Monosialyllacto-*N*-hexaose	0.11 (0.05, 0.16)	0.16 (0.10, 0.31)
4021a + S-LNnH II*	4201	No literature name + sialyllacto-*N*-neohexaose II	0.56 (0.33, 0.89)	0.35 (0.16, 0.70)
MFpLNH IV*	4210	Fucosyl-*para*-lacto-*N*-hexaose	2.8 (2.2, 3.4)	3.3 (2.4, 3.9)
4120a*	4210	No literature name	0.08 (0.04, 0.27)	0.14 (0.05, 0.57)
MFLNH I + III*	4210	Monofucosyllacto-*N*-hexaose I + III	2.5 (1.6, 3.3)	3.3 (2.2, 4.9)
IFLNH III*	4210	Isomer 3 fucosyl-*para*-lacto-*N*-hexaose	1.8 (1.2, 2.2)	1.5 (0.7, 2.3)
IFLNH I*	4210	Isomer 1 fucosyl-*para*-lacto-*N*-hexaose	0.24 (0.07, 0.75)	0.06 (0.03, 0.16)
4211a^8^	4211	No literature name	0.21 (0.07, 0.32)	0.35 (0.12, 0.40)
4211b*	4211	No literature name	0.12 (0.07, 0.19)	0.29 (0.15, 0.31)
4211c*	4211	No literature name	1.9 (1.5, 2.3)	2.4 (1.9, 3.0)
DFLNHa*	4220	Difucosyllacto-*N*-hexaose (a)	0.82 (0.36, 1.84)	0.10 (0.05, 0.15)
DFLNHb*	4220	Difucosyllacto-*N*-hexaose	1.33 (0.83, 2.01)	4.3 (3.0, 5.7)
DFLNHc*	4220	Difucosyllacto-*N*-hexaose (c)	0.11 (0.05, 0.21)	0.06 (0.03, 0.19)
DFpLNH II*	4220	Difucosyl-*para*-lacto-*N*-hexaose	1.9 (1.4, 2.3)	2.2 (1.6, 2.9)
DFS-LNnH*	4221	Difucosylmonosialyllacto-*N*-neohexaose	0.03 (0.04, 0.13)	0.03 (0.04, 0.05)
TFLNH*	4230	Trifucosyllacto-*N*-hexaose	1.01 (0.11, 1.37)	0.7 (0.1, 1.0)
4240a*	4240	No literature name	0.11 (0.04, 0.22)	0.03 (0.02, 0.04)
4320a*	4320	No literature name	0.10 (0.05, 0.19)	0.08 (0.04, 0.18)
5130a*	5130	No literature name	0.34 (0.15, 0.61)	0.45 (0.24, 0.89)
5130b*	5130	No literature name	0.09 (0.05, 0.19)	0.11 (0.06, 0.18)
5130c*	5130	No literature name	0.10 (0.04, 0.27)	0.07 (0.02, 0.21)
5230a + DFLNnO I/DFLNO II*	5230	Difucosyllacto-*N*-neooctaose I/difucosyllacto-*N*-octaose II + 5230	0.76 (0.45, 0.98)	0.56 (0.23, 1.04)
5230a*	5230	No literature name	0.17 (0.04, 0.34)	0.18 (0.04, 0.40)
5230b	5230	No literature name	0.14 (0.08, 0.22)	0.17 (0.10, 0.29)
5300a	5300	No literature name	0.47 (0.20, 0.78)	0.30 (0.14, 0.65)
F-LNO*^9^	5310	Fucosyllacto-*N*-octaose	0.54 (0.33, 0.74)	0.54 (0.31, 0.89)
5311a	5311	No literature name	0.05 (0.03, 0.09)	0.05 (0.03, 0.10)
DFLNO I*	5320	Difucosyllacto-*N*-octaose I	0.31 (0.15, 0.62)	0.84 (0.26, 1.30)
DFLNnO II*	5320	Difucosyllacto-*N*-neooctaose II	0.23 (0.10, 0.44)	0.34 (0.08, 0.77)
DFLNnO I/DFLNO II*	5320	Difucosyllacto-*N*-neooctaose I/difucosyllacto-*N*-octaose II	0.44 (0.09, 0.79)	0.13 (0.05, 0.53)
5330a	5330	No literature name	0.04 (0.03, 0.15)	0.05 (0.03, 0.06)
6400a^10^	6400	No literature name	0.03 (0.02, 0.06)	0.03 (0.02, 0.05)
6400b	6400	No literature name	0.05 (0.04, 0.11)	0.05 (0.03, 0.07)

1 ARI, acute respiratory infection; CRP, C-reactive protein; *FUT2*, fucosyltransferase 2; HMO, human milk oligosaccharide.

^2*^Median values differed between secretors and nonsecretors.

^3^Composition given as hexose_*N*-acetylhexoseamine (HexNAc)_fucose_*N*-acetylneuraminic acid (sialic acid). For example, 5311 has 5 hexoses, 3 HexNAc, 1 fucose, and 1 sialic acid. Thus, those with a nonzero number in the last position are sialylated; those with a nonzero number in the third position are fucosylated.

4 Positive for the functional enzyme encoded by the *FUT2* gene.

5 Values are median (25th, 75th percentile).

6 There was an interaction between F-LSTc and secretor status for longitudinal prevalence of diarrhea from 6 to 7 mo.

7 There were interactions between LNH and secretor status for longitudinal prevalence of diarrhea and lost appetite from 6 to 7 mo.

8 There were interactions between the unnamed HMO 4211a and secretor status for CRP at 18 mo and prevalence of elevated CRP at 18 mo.

9 There were interactions between F-LNO and secretor status for incidence of diarrhea from 6 to 7 mo, and incidence of ARI and lost appetite from 6 to 12 mo.

10 There were interactions between the unnamed HMO 6400a and secretor status for incidence of any illness, fever, and lost appetite from 6 to 12 mo; longitudinal prevalence of fever and lost appetite from 6 to 12 mo; and high CRP at 18 mo.

All statistical analyses were performed using SAS version 9.4 (SAS Institute Inc.). We first examined whether median values of HMOs and proteins differed between women with the secretor phenotype [positive for the functional enzyme encoded by the fucosyltransferase 2 (*FUT2*) gene, as determined by >6% α1-2-linked fucosylated HMOs, as described previously (22)] and nonsecretors using the nonparametric Kruskal–Wallis test. Covariate adjusted regression models were examined separately in secretors and in nonsecretors for those HMOs that differed between secretors and nonsecretors, and also when the interaction between secretor status and the predictor variable was significant. Otherwise, secretors and nonsecretors were analyzed together. To explore associations of the relative abundance of α1-2-fucosylated HMOs with morbidity and inflammation, regression models were examined in all women, as well as separately in secretors and nonsecretors.

Spearman correlation coefficients were used to assess associations of the bioactive proteins with groups of HMOs and individual HMOs. Negative binomial models were used to examine associations of HMOs and bioactive proteins with continuous morbidity outcomes. Negative binomial models were used because of the nonnormal distribution of morbidity data. The incidence of fever and diarrhea from 6 to 7 mo was too low for models to estimate, so binary variables were created for those variables and associations were examined using logistic regression. Associations between HMOs/proteins and CRP and AGP were examined using linear regression for the continuous outcome variables, and logistic regression for binary outcome variables. All models were adjusted for covariates. Covariates included baseline maternal age, height, BMI, parity, education, food security, HIV status, hemoglobin, household assets, and residential location, as well as infant sex and season at the time of sample collection, and intervention group. For the morbidity associations, the number of days morbidity data were available for each symptom was included as an offset in the models. Also, we excluded children from the morbidity analyses if we had data for fewer than half of the days during the follow-up time interval for that child. For all regression models the residuals were checked for normality and homoskedasticity was evaluated via Q-Q plots, Shapiro–Wilk testing, and Breusch–Pagan testing. We adjusted all models of the primary analyses for multiple hypothesis tests by using the Benjamini–Hochberg procedure (36), using a false discovery rate of 15%. Groups for the Benjamini–Hochberg procedure were formed based on the outcome variable. We did not adjust for multiple hypothesis testing for the exploratory analyses.

As an exploratory analysis we conducted a factor analysis to determine whether groups of HMOs were associated with the outcome variables. We first assessed whether there were combinations of HMOs that represented a latent factor or group. We then analyzed associations between each factor and the outcomes. Factor analysis was performed using iterated principal axis factor analysis with a varimax rotation. We used a factor loading cutoff of 0.5 to describe the principal HMOs contributing to a specific factor.

## Results

### Participant information

Of the 1391 women enrolled in the study, 869 were assigned to the complete intervention and followed up to 18 mo after delivery. Of those, we collected breast milk from 659 at 6 mo postpartum. From those samples, we successfully analyzed HMOs from 647 and proteins from 637 samples (**Supplemental Figure 1)**. The mean (±SD) maternal age at study enrollment was 25.0 ± 6.0 y; the mean maternal BMI at study enrollment was 22.0 ± 2.7 kg/m^2^; the median (25th, 75th percentile) maternal years of formal education was 3 (0, 6) y; and 47.3% of infants were males.

Infant morbidity data were available for 555 participants (84%) from 6 to 7 mo, and 587 participants (89%) from 6 to 12 mo. The incidence and longitudinal prevalence of illnesses within each follow-up period are presented in **Table 3**. CRP and AGP were available for 490 of parti-cipants (74%) at 6 mo and 528 (80%) at 18 mo of age. The median (25th, 75th percentile) CRP was 2.1 (0.5, 6.5) mg/L at 6 mo, and 2.0 (0.6, 7.4) mg/L at 18 mo. The median (25th, 75th percentile) AGP was 1.2 (0.9, 1.5) g/L at 6 mo, and 1.3 (0.9, 1.6) g/L at 18 mo.

**TABLE 3:** Incidence and longitudinal prevalence of morbidity in Malawian children from 6 to 7 mo (*n* = 551) and 6 to 12 mo (*n* = 583)

Illness	Timeframe	Percentage of children without illness	Incidence^1^**^,^**^2^	Longitudinal prevalence^2^**^,^**^3^
Any illness	6 to 7 mo	41	3.6 (0, 6.9)	9.7 (0, 25.8)
	6 to 12 mo	3	3.4 (1.9, 5.7)	12.9 (6.4, 23.8)
Fever	6 to 7 mo	69	0 (0, 3.4)	0 (0, 6.2)
	6 to 12 mo	24	1.1 (0.5, 2.2)	3.4 (0.6, 7.2)
Diarrhea	6 to 7 mo	84	0 (0, 0)	0 (0, 0)
	6 to 12 mo	41	0.6 (0, 1.7)	1.3 (0, 4.5)
Acute respiratory infection	6 to 7 mo	51	0 (0, 4.8)	3.2 (0, 22.6)
	6 to 12 mo	7	2.4 (1.2, 4.0)	9.4 (4.0, 17.5)
Lost appetite	6 to 7 mo	83	0 (0, 0)	0 (0, 0)
	6 to 12 mo	39	0.6 (0, 1.3)	1.7 (0, 5.0)

1 The number of new episodes of symptoms that followed ≥2 symptom-free days within the follow-up period.

2 Values are median (25th, 75th percentile).

3 The percentage of days of illness symptoms within the days morbidity data were available for each symptom.

In this population, 75% of mothers had the secretor phenotype. The relative abundance of all groups of HMOs (fucosylated, sialylated, fucosylated and sialylated, and nonfucosylated neutrals) differed between maternal secretors and nonsecretors, as did that of the individual HMOs marked with asterisks in Table 2. The content of the 6 bioactive milk proteins did not differ between maternal secretors and nonsecretors. Interactions between proteins or HMOs and secretor status for infant morbidity and inflammation are noted in Tables 1 and 2.

There were several significant associations of the bioactive breast milk proteins with the other bioactive proteins, groups of HMOs, or individual HMOs (**Supplemental Table 1**) as described previously (22).

Many associations of HMOs and bioactive proteins with infant morbidity and markers of inflammation were significant, as described below. For the significant associations among the exploratory analyses (group of HMOs that are both fucosylated and sialylated, group of neutral, nonfucosylated HMOs, and individual HMOs), the outcome values at the 1st and 5th quintiles of the predictor HMO or group of HMOs are presented in **Supplemental Table 2**.

### Associations of milk constituents with infant morbidity

#### Infant morbidity in infants of all women

For the morbidity outcomes, among the HMOs and bioactive proteins that did not differ in content between secretors and nonsecretors, and for which there was no significant interaction with secretor status (**Figure 1**A), there were no significant associations for the primary analyses. There was a mix of positive, negative, and null associations for the exploratory analyses: for 3 of the 51 individual HMOs there were positive associations with ≥1 of the morbidity outcomes, and for another 2 HMOs there were negative associations. None of the associations had a *P* value <0.01. There were no significant associations between α1-2-fucosylated HMOs and any of the morbidity outcomes in all women combined.

**FIGURE 1:**
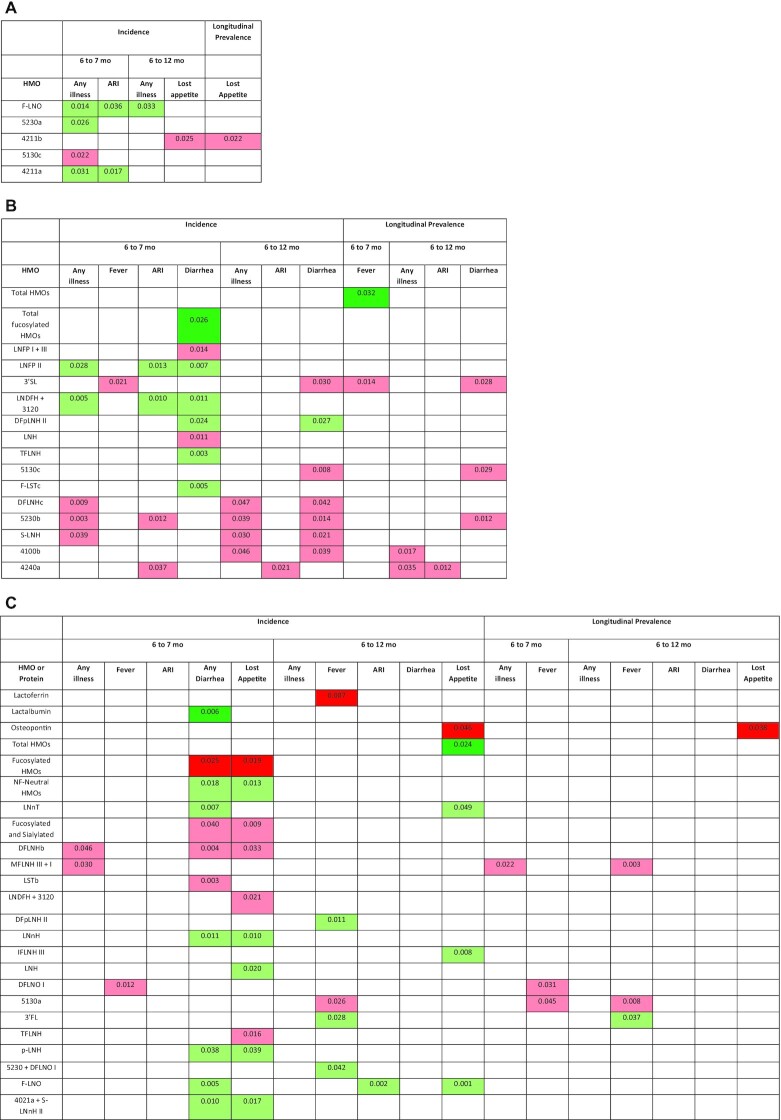
*Continued*.

**FIGURE 1:**
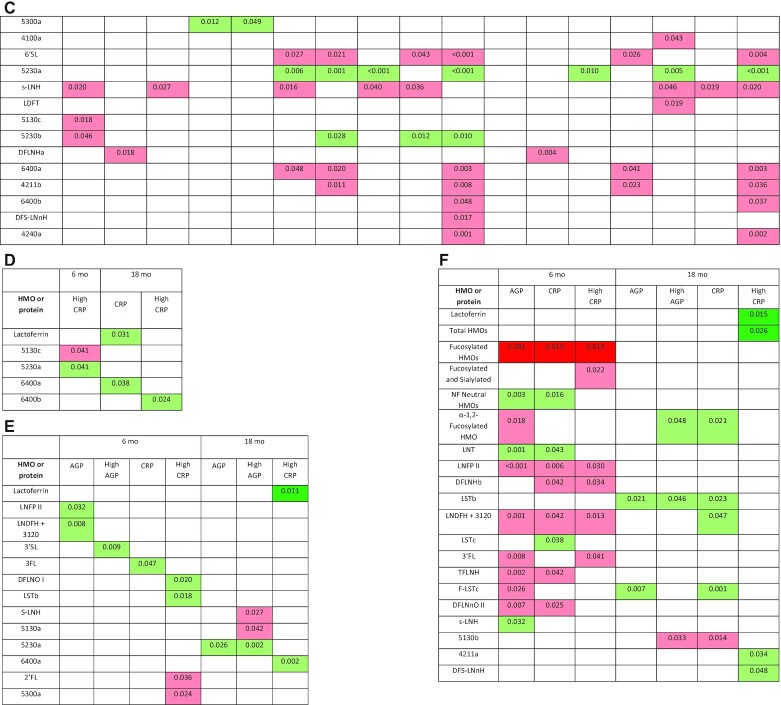
Associations of human milk oligosaccharides (HMOs) and bioactive proteins at 6 mo with infant morbidity and markers of inflammation in 659 Malawian mother-infant dyads: morbidity outcomes in all participants (A) for milk constituents that did not differ between secretors (positive for the functional enzyme encoded by the *FUT2* gene) and nonsecretors; morbidity outcomes in secretors (B) and nonsecretors (C) for milk constituents that differed between secretors and nonsecretors, or for which there was a significant interaction between the milk constituent and secretor status; markers of inflammation in all participants (D) for milk constituents that did not differ between secretors and nonsecretors; markers of inflammation in secretors (E) and nonsecretors (F) for milk constituents that differed between secretors and nonsecretors, or for which there was a significant interaction between the milk constituent and secretor status. Only shown are the milk constituents that were significantly associated with ≥1 of the outcomes. HMOs are ordered from highest to lowest relative abundance. Associations of outcomes with the bioactive proteins, total abundance of HMOs, and relative abundance of fucosylated HMOs and sialylated HMOs were considered primary analyses. All others were considered exploratory analyses. For the associations of morbidity outcomes, *P* values were determined by negative binomial models using continuous morbidity data for all associations except incidence of fever and diarrhea from 6 to 7 mo, which were calculated using logistic regression with binary morbidity data. For the associations of markers of inflammation, *P* values were determined by multiple linear regression models for continuous AGP and CRP data and logistic regression for binary outcomes. *P* values are from models that were adjusted for covariates, with a level of significance <0.05. Associations in dark green are primary analyses that were in the positive direction and remained significant after the Benjamini–Hochberg correction for multiple hypothesis testing. Associations in light green are exploratory HMOs that were in the positive direction or primary analyses that did not remain significant after correcting for multiple hypothesis testing. Associations in pink are exploratory HMOs that were in the negative direction. Associations in red are primary analyses that were in the negative direction and remained significant after the Benjamini–Hochberg procedure. HMOs identified by numbers have not been previously named. Their composition is given as hexose_*N*-acetylhexoseamine (HexNAc)_fucose_*N*-acetylneuraminic acid (sialic acid). For example, 5311 has 5 hexoses, 3 HexNAc, 1 fucose, and 1 sialic acid. Thus, those with a nonzero number in the last position are sialylated; those with a nonzero number in the third position are fucosylated. AGP, α-1-acid glycoprotein; ARI, acute respiratory infection; CRP, C-reactive protein; DFLNH(a,b,c), difucosyllacto-*N*-hexaose (a,b,c isomers); DFLNnO, difucosyllacto-*N*-neooctaose; DFLNO, difucosyllacto-*N*-octaose; DFpLNH, difucosyl-*para*-lacto-*N*-hexaose; DFS-LNnH, difucosylmonosialyllacto-*N*-neohexaose; F-LNO, fucosyllacto-*N*-octaose; F-LST, fucosyl-sialyllacto-*N*-tetraose; *FUT2*, fucosyltransferase 2; HMO, human milk oligosaccharide; IFLNH, fucosyl-*para*-lacto-*N*-hexaose; LDFT, lactodifucotetraose; LNDFH, lacto-*N*-difucohexaose; LNFP, lacto-*N*-fucopentaose; LNH, lacto-*N*-hexaose; LNnH, lacto-*N*-neothexaose; LNnT, lacto-*N*-neotetraose; LNT, lacto-*N*-tetraose; LST(a,b,c), sialyllacto-*N*-tetraose(a,b,c isomer); MFLNH, monofucosyllacto-*N*-hexaose; MFpLNH, fucosyl-*para*-lacto-*N*-hexaose; NF, non-fucosylated; p-LNH, *para*-lacto-*N*-hexaose; s-LNH, monosialyllacto-*N*-hexaose; S-LNnH, sialyllacto-*N*-neohexaose; TFLNH, trifucosyllacto-*N*-hexaose; 2′FL, 2′-fucosyllactose; 3′FL, 3′-fucosyllactose; 3′SL, 3′-sialyllactose; 6′SL, 6′-sialyllactose.

#### Infant morbidity in infants of secretors only

In secretors only (for the HMOs and proteins that were either different between secretors and nonsecretors or had an interaction with secretor status; Figure 1B) there were positive associations between total absolute abundance of HMOs and longitudinal prevalence of fever from 6 to 7 mo (*P* = 0.032), and between relative abundance of fucosylated HMOs and incidence of diarrhea from 6 to 7 mo (*P*  = 0.026) after adjusting for multiple hypothesis testing. Specifically, for every one-tenth of a unit increase in absolute abundance of HMOs, the odds of the child having fever from 6 to 7 mo increased by 3.1% (95% CI: 0.2% to 7.0%); for every 0.1% increase in relative abundance of fucosylated HMOs, the odds of the child having diarrhea from 6 to 7 mo increased by 16.1% (95% CI: 10.6% to 24.3%). There was no difference in the percentage of infants with diarrhea from 6 to 7 mo between those whose mothers had a relative abundance of fucosylated HMOs above compared with below the mean of 60.3%.

For the exploratory analyses in secretors, there were positive, negative, and null associations: for 5 of the individual HMOs there were positive associations with ≥1 of the morbidity outcomes [those with *P* values <0.01 included lacto-*N*-fucopentaose II (LNFP II), lacto-*N*-difucohexaose I + II + unknown 3120 (LNDFH + 3120), and trifucosyllacto-*N*-hexaose (TFLNH)], and for 9 of the individual HMOs there were negative associations with ≥1 of the morbidity outcomes [those with *P* values <0.01 included difucosyllacto-*N*-hexaose (c) (DFLNHc), and the unnamed HMOs 5130c and 5230b]. There were no significant associations between α1-2-fucosylated HMOs and any of the morbidity outcomes in secretors.

#### Infant morbidity in infants of nonsecretors only

**FIGURE 2:**
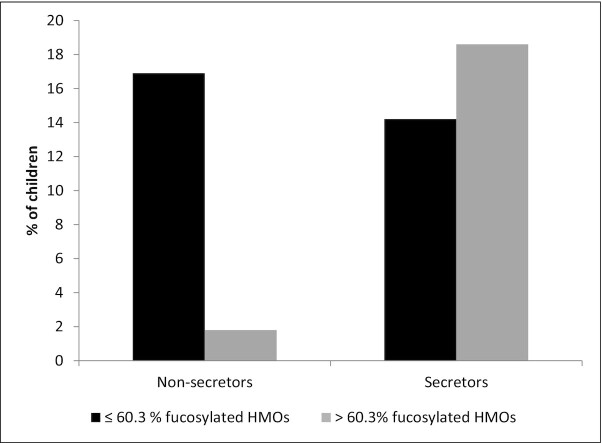
Percentage of Malawian children who had any diarrhea from 6 to 7 mo, by mother's secretor status (positive for the functional enzyme encoded by the *FUT2* gene) and relative abundance of fucosylated human milk oligosaccharides (HMOs). The mean relative abundance of fucosylated HMOs in all women was selected as the cutoff. In nonsecretors there was a significant difference in the percentage of children with diarrhea between those with low and high relative abundance of fucoslyated HMOs (*P* = 0.005, analyzed by using generalized linear models). *FUT2*, fucosyltransferase 2.

In nonsecretors only (for the HMOs and proteins that were either different between secretors and nonsecretors or had an interaction with secretor status) there were several significant associations between the milk constituents and morbidity outcomes, many of which remained significant after adjusting for multiple hypothesis testing (Figure 1C). There were positive associations between lactalbumin and incidence of diarrhea from 6 to 7 mo (*P* = 0.006), and between absolute abundance of HMOs and incidence of lost appetite from 6 to 12 mo (*P*  = 0.024). For every 0.1 g/L increase in lactalbumin concentration, the odds of diarrhea from 6 to 7 mo were 32.2% (95% CI: 14.0% to 74.1%) higher; for every one-tenth of a unit increase in absolute abundance of HMOs, the odds of lost appetite from 6 to 12 mo were 4.2% (95% CI: 0.4% to 9.2%) higher. There were inverse associations between lactoferrin and incidence of fever from 6 to 12 mo (*P* = 0.007); between osteopontin and incidence and longitudinal prevalence of lost appetite from 6 to 12 mo (*P* = 0.046 and *P* = 0.038, respectively); and between relative abundance of fucosylated HMOs and incidence of diarrhea and lost appetite from 6 to 7 mo (*P* = 0.025 and *P*  = 0.019). For every 0.1 g/L increase in lactoferrin concentration, the odds of fever incidence from 6 to 12 mo were 2.7% (95% CI: 0.8% to 4.2%) lower; for every one-tenth of a unit increase in osteopontin, the odds of incidence and longitudinal prevalence of lost appetite from 6 to 12 mo were lower by 2.6% (95% CI: 0.04% to 4.6%) and 3.5% (95% CI: 0.3% to 5.7%), respectively; and for every 0.1% increase in relative abundance of fucosylated HMOs, the incidence of diarrhea and lost appetite from 6 to 7 mo was lower by 4.8% (95% CI: 0.8% to 7.0%) and 4.9% (95% CI: 1.1% to 7.1%), respectively. The percentage of infants with diarrhea from 6 to 7 mo was greater in those of mothers who secreted lower compared with higher relative abundance of fucosylated HMOs (*P* = 0.005; **Figure 2**).

For the exploratory analyses in nonsecretors, there were positive, negative, and null associations: the undecorated (nonfucosylated neutral) HMOs as well as 12 of the individual HMOs were positively associated with ≥1 morbidity outcomes [those with *P* values <0.01 included lacto-*N*-neotetraose, fucosyl-*para*-lacto-*N*-hexaose (IFLNH III), fucosyllacto-*N*-octaose (F-LNO), and the unnamed HMO 5230a], and 20 of the individual HMOs were negatively associated with ≥1 morbidity outcomes [those with *P* values <0.01 included DFLNHb, DFLNHa, monofucosyllacto-*N*-hexaose I + III (MFLNH III + I), sialyllacto-*N*-tetraose (b) (LSTb), 6′-sialyllactose, and the unnamed HMOs 5130a, 6400a, 4211b, and 4240a]. There were no significant associations between α1-2-fucosylated HMOs and any of the morbidity outcomes in nonsecretors.

### Associations of milk constituents with markers of inflammation

#### Markers of inflammation in infants of all women

For the markers of inflammation, in secretors and nonsecretors combined there were no significant associations for the primary analyses, other than a positive association between lactoferrin and CRP at 18 mo (*P* = 0.031) that did not remain significant after adjusting for multiple hypothesis testing (Figure 1D). There was a mix of positive, negative, and null associations among the exploratory analyses: 6 of the HMOs were positively associated with CRP at either 6 or 18 mo, whereas 1 of the HMOs was inversely associated with CRP at either 6 or 18 mo. None of the associations had a *P* value <0.01. There were no significant associations between α1-2-fucosylated HMOs and any of the markers of inflammation in all women combined.

#### Markers of inflammation in infants of secretors only

In secretors only, there was a positive association between lactoferrin at 6 mo and elevated CRP at 18 mo that remained significant after multiple hypothesis testing (*P* = 0.011; Figure 1E). For every 0.1 g/L increase in lactoferrin concentration, the odds of high CRP at 18 mo were 3.6% (0.7% to 7.2%) higher. There were no other significant associations among the primary analyses. Among the exploratory analyses there were several positive, negative, and null associations: 9 of the individual HMOs were positively associated with ≥1 marker of inflammation [those with *P* values <0.01 included 3′-sialyllactose (3′SL), LNDFH + 3120, and the unnamed HMOs 5230a and 6400a], whereas 4 HMOs were inversely associated with ≥1 of the markers of inflammation, none of which had a *P* value <0.01. There were no significant associations between α1-2-fucosylated HMOs and any of the markers of inflammation in secretors.

#### Markers of inflammation in infants of nonsecretors only

In nonsecretors only, there were inverse associations between the relative abundance of fucosylated HMOs and AGP, CRP, and elevated CRP at 6 mo (*P* = 0.001, *P* = 0.010, and *P* = 0.019, respectively; Figure 1F), all of which remained significant after adjusting for multiple hypothesis testing. There were positive associations between lactoferrin at 6 mo and elevated CRP at 18 mo (*P* = 0.015), and between absolute abundance of HMOs at 6 mo and elevated CRP at 18 mo (*P* = 0.026), both of which remained significant after multiple hypothesis testing. For every 0.1 g/L increase in lactoferrin concentration, the odds of high CRP at 18 mo were 8.5% (1.3% to 30.4%) higher; for every one-tenth of a unit increase in value of absolute abundance of HMOs, the odds of high CRP at 18 mo were 7.8% (0.7% to 29.6%) higher. The group of HMOs that were both fucosylated and sialylated, as well as 17 individual HMOs, were inversely associated with ≥1 markers of inflammation [those with a *P* value <0.01 included LNFP II, LNDFH + 3120, 3′fucosyllactose (3′FL), TFLNH, and difucosyllacto-*N*-neooctaose II]. The undecorated HMOs, as well as 12 of the individual HMOs were positively associated with ≥1 of the markers of inflammation [the group of undecorated HMOs, as well as lacto-*N*-tetraose (LNT) and monofucosylmonosialyllacto-*N*-neotetraose had *P* values <0.01]. In nonsecretors, there were positive associations between α1-2-fucosylated HMOs and CRP at 18 mo (*P* = 0.021) and high AGP at 18 mo (*P* = 0.048), and a negative association between α1-2-fucosylated HMOs and AGP at 6 mo (*P* = 0.018).

### Factor analysis

The factor analysis resulted in 1 group of HMOs (MFLNH III + I, difucosyllacto-*N*-octaose I, monosialyllacto-N-hexaose, and the unnamed HMO 5130a—all relatively large decorated HMOs) that were inversely associated with incidence of any illness from 6 to 7 mo (expected count ratio: 0.86; 95% CI: 0.76, 0.97; *P* = 0.014), and tended toward an inverse association with incidence of diarrhea from 6 to 7 mo (0.71; 95% CI: 0.50, 1.001; *P* = 0.051). Neither this factor nor any other factors were associated with other morbidity outcomes.

The factor analysis did not reveal any significant association with markers of inflammation.

## Discussion

We tested the overall hypothesis that breast milk content of bioactive proteins and HMOs is inversely associated with infant morbidity and markers of inflammation. In nonsecretors we found several inverse associations that supported our hypotheses—for example, between *1*) lactoferrin and fever; *2*) osteopontin and loss of appetite; and *3*) relative abundance of fucosylated HMOs and diarrhea, loss of appetite, and markers of inflammation—but also several positive associations that were in the opposite direction of our hypotheses—for example, between *1*) total abundance of HMOs and both lost appetite and elevated CRP; *2*) lactalbumin and diarrhea; and *3*) lactoferrin and elevated CRP. In secretors we found a few positive associations that contradicted our hypotheses (e.g., between total abundance of HMOs and fever, and between lactoferrin and elevated CRP). Interestingly, we found an inverse association between relative abundance of fucosylated HMOs and diarrhea in nonsecretors, but a positive association in secretors. Similarly, the relative abundance of fucosylated HMOs was inversely associated with inflammation in nonsecretors, but not in secretors. We also explored associations between individual HMOs and infant outcomes and found several that were noteworthy, most of which were in nonsecretors and most of which were inverse associations.

Inverse associations between the relative abundance of fucosylated HMOs and infant diarrhea and inflammation, yet lack of associations between α1-2-fucosylated HMOs and morbidity and markers of inflammation suggest that the combination of both α1-2- and non–α1-2-fucosylated HMOs contributes to reduced morbidity and inflammation. Others have reported inverse associations between α1-2-fucosylated HMOs and diarrhea, yet in those studies only 4 α1-2-fucosylated HMOs and 2 non–α1-2-fucosylated HMOs were examined (9, 37). In our analyses, the inverse associations we found were in nonsecretors who produce very little α1-2-fucosylated HMOs (38), suggesting that the apparent protection against diarrhea in nonsecretors likely comes from fucosylated HMOs other than those with α1-2-linked fucoses.

In nonsecretors there were inverse associations between milk lactoferrin concentration and infant fever, and between milk osteopontin and lost appetite. We could find no other studies that reported associations between lactoferrin and fever. Lactoferrin has been associated with fewer lower respiratory tract infections (39) and decreased longitudinal prevalence, severity, and duration of diarrhea (40). We could find no studies that reported associations between osteopontin and anorexia, although Lönnerdal and colleagues (41) reported lower TNF-α in infants fed formula fortified with osteopontin compared with standard formula. TNF-α is known to cause anorexia (42). Thus, the inverse association that we observed with lost appetite could be due to lower concentrations of TNF-α in infants of mothers with higher milk osteopontin concentrations. Lönnerdal and colleagues (41) also found a lower incidence of fever in the osteopontin group than the standard formula group. We did not find an association between osteopontin and incidence or longitudinal prevalence of fever.

Only a few other studies have examined associations of specific HMOs and bioactive proteins with infant morbidity and inflammation. In a small sample of mother-infant pairs from the Gambia (*n* = 33), Davis and colleagues (21) found that mothers of infants who were sick produced milk with lower concentrations of LNFP I + III than mothers of infants who were not sick. We found a similar association between LNFP I + III and diarrhea from 6 to 7 mo, though only in secretors (we were unable to chromatographically separate LNFP I and LNFP III during analysis, although LNFP III is <5% of the abundance of LNFP I). Davis and colleagues also found a higher relative abundance of F-LNO in mothers of sick infants. We found positive associations between F-LNO and incidence of any illness from 6 to 7 mo and 6 to 12 mo, as well as incidence of acute respiratory infection from 6 to 7 mo in secretors and nonsecretors combined. In nonsecretors only, we found positive associations between F-LNO and incidence of diarrhea from 6 to 7 mo, and incidence of ARI and lost appetite from 6 to 12 mo. Davis also reported higher IFLNH III in mothers of sick infants, whereas we found a positive association between IFLNH III and incidence of lost appetite from 6 to 12 mo. It is noteworthy that F-LNO and IFLNH III have α1-3-linked fucoses, whereas LNFP I + III has mostly α1-2-, as well as some α1-3-linked fucoses. Higher concentrations of α1-2-linked fucosylated HMOs have been shown previously to be associated with decreased incidence of diarrhea compared with higher concentrations of non–α1-2-linked fucosylated HMOs (37).

We found several positive associations of particular bioactive milk constituents with infant morbidity and markers of inflammation. Many of these associations could be spurious, particularly those with relatively high *P* values. However, there are a few possible explanations for the positive associations, one of which is that that some milk constituents could be produced in response to illness, rather than acting to prevent an infection. By using ultrasound, researchers found a retrograde or reverse ductal flow from baby to mother that could allow immune secretions or pathogens in the infant's saliva (from gastrointestinal or respiratory secretions) to pass back into the collecting ducts of the mammary gland and initiate a maternal response (43, 44). Breakey and colleagues (45) found increased concentration of lactoferrin in breastmilk of Argentinian mothers of sick infants, whereas Riskin and colleagues (46) found that when the breastfeeding baby, but not mother, was sick, there was a trend toward increased lactoferrin concentration (*P* = 0.072) and significantly higher concentrations of macrophages, lymphocytes, neutrophils, and CD45+ cells in breast milk. It is possible that such a retrograde stimulation of maternal production of these constituents has occurred in our study population.

Another possible explanation for the positive associations of milk constituents with morbidity and inflammation is a physical interaction between the constituents and pathogens that increases their infectivity. Ramani and colleagues (47) found higher concentrations of 2′-fucosyllactose, 6′-sialyllactose, and LNT in milk from Indian mothers of rotavirus-positive neonates with gastrointestinal symptoms compared with those of rotavirus-negative neonates. The authors proposed that the higher concentrations of some HMOs in mothers of rotavirus-positive neonates could be due to structural stability conferred by HMOs to pathogens, similar to the stabilization of poliovirus by the polysaccharide component of LPS (48), or alternatively, the pathogens could utilize a cellular entry pathway intended for HMO transport to gain entry into cells. The authors do not mention the possible retrograde stimulation of HMO production, but if retrograde stimulation of milk constituent production exists, it is feasible that the presence of those bacteria stimulated the production of particular HMOs.

This study had several strengths, including the large number of HMOs and bioactive proteins that we were able to analyze, the relatively large sample size, which allowed us ample power to analyze associations separately in secretors and nonsecretors while controlling for multiple covariates, the longitudinal study design, which allowed us to follow the infants to determine short- and long-term associations with morbidity and inflammation, and analyzing data only from breast milk samples collected within 2 wk of the scheduled 6-mo study visit, which omitted the potentially confounding influence of lactation stage on breast milk concentration of bioactive breast milk components (21, 49, 50). The main limitation of the study was that the analyses were conducted in 2015 when few HMO or osteopontin standards were available or only available at a prohibitive cost, which led us to examine associations using HMO and osteopontin ion counts rather than concentration. However, the intent of this study was to examine associations with infant outcomes, rather than examine quantities of HMOs. We believe that using relative abundances based on ion abundances is sufficient and allowed us to examine associations of 51 HMOs with the outcomes. Had we used concentrations, we would have been limited to analyzing associations among only 5 or so HMOs. We were further limited by not having data on quantity of breast milk consumed, which prevents us from assessing associations of total infant intake of these breast milk constituents with infant morbidity and inflammation. However, the associations of HMOs with infant growth were found to be similar whether the exposure was defined as HMO concentration or total intake (24). Another limitation was that we analyzed breast milk constituents from samples collected at 1 time point, so we might have missed associations among constituents that were transiently low or high at the time of sample collection, due to intraindividual (diurnal or day-to-day) variation. Lastly, we were limited by relying on caregiver reporting of morbidity outcomes, which could be biased compared with direct assessments.

In summary, several HMOs and bioactive proteins were associated with infant morbidity and markers of inflammation, with most of these associations observed in nonsecretors. Although many of the associations supported our hypotheses of inverse associations, there were several unexpected positive associations. Further research is needed to elucidate why most of the associations were only seen in nonsecretors, to determine whether infant histo-blood group antigen secretor status modifies associations of milk constituents with morbidity and inflammation, and to determine whether a causal relation exists between HMOs or bioactive proteins and infant morbidity and inflammation, and if so, the direction of causality.

## Supplementary Material

nzab072_Supplemental_File

## Notes

This publication is based on research funded in part by grants from the Bill & Melinda Gates Foundation to the University of California, Davis and Washington University, St Louis.

Data described in the manuscript, code book, and analytic code will be made available upon request pending approval by all coauthors.

Author disclosures: The authors report no conflicts of interest.

Supplemental Tables 1 and 2 and Supplemental Figure 1 are available from the “Supplementary data” link in the online posting of the article and from the same link in the online table of contents at https://academic.oup.com/cdn/.

Abbreviations used: AGP, α-1-acid glycoprotein; ARI, acute respiratory infection; CRP, C-reactive protein; DFLNH, difucosyllacto-*N*-hexaose; F-LNO, fucosyllacto-*N*-octaose; HHAZ, household asset *z*-score; HMO, human milk oligosaccharide; IFLNH, fucosyl-*para*-lacto-*N*-hexaose; LNDFH, lacto-*N*-difucohexaose; LNFP, lacto-*N*-fucopentaose; LNS, lipid-based nutrient supplement; LNT, lacto-*N*-tetraose; LST, sialyllacto-*N*-tetraose; MFLNH, monofucosyllacto-*N*-hexaose; TFLNH, trifucosyllacto-*N*-hexaose; 3′-fucosyllactose; 3′SL, 3′-sialyllactose.
