# Supplementary material for: Associations of Human Milk Oligosaccharides and Bioactive Proteins with Infant Morbidity and Inflammation in Malawian Mother-Infant Dyads
Source: Curr Dev Nutr. 2021 Apr 29;5(5):nzab072. doi: 10.1093/cdn/nzab072 (PMC8163417; doi:10.1093/cdn/nzab072)
Supplement: nzab072_Supplemental_File [file nzab072_supplemental_file.pdf]

Associations of human milk oligosaccharides and bioactive proteins with infant morbidity and inflammation among Malawian mother-infant dyads.

Josh M Jorgensen, et al.

Online Supplementary Material

Supplementary Table 1. Spearman's correlation coefficients for associations of bioactive proteins with human milk oligosaccharides among Malawian women at 6 mo postpartum. Due to sizing issues, the table is separated into smaller tables. DFLNH, difucosyllacto-N-hexaose; DFLNnO, difucosyllacto-N-neooctaose; DFS-LNnH, difucosylmonosialyllacto-N-neohexaose; F-LST, fucosyl-sialyllacto-N-tetraose; IFLNH, fucosyl-para-lacto-N-hexaose; LDFT, lactodifucotetraose; LNDFH, lacto-N-difucohexaose; LNFP, lacto-N-fucopentaose; LNH, lacto-N-hexaose; LNnH, lacto-N-neothexaose; LNnT, lacto-N-neotetraose; LNT, lacto-N-tetraose; LST, sialyllacto-N-tetraose; MFpLNH IV, fucosyl-para-lacto-N-hexaose; p-LNH, para-lacto-N-hexaose; S-LNnH, sialyllacto-N-neohexaose; TFLNH, trifucosyllacto-N-hexaose; 2'FL, 2'-fucosyllactose; 3'FL, 3'-fucosyllactose; 3'SL, 3'-sialyllactose; 6'SL, 6'-sialyllactose. HMOs identified by numbers have not been previously named. Their composition is given as Hexose_N-acetylhexoseamine (HexNAc)_Fucose_N-Acetylneuraminic acid (sialic acid). For example, 5311 has 5 Hexoses, 3 HexNAc, 1 fucose, and 1 sialic acid. Thus, those with a non-zero number in the last position are sialylated; those with a non-zero number in the 3rd position are fucosylated.

		Absolute abundance HMOs	%Fucosylated HMOs	%Sialylated HMOs	%Fucosylated or sialylated HMOs	%Non-fucosylated neutral HMOs	%3FL	%2FL	%LDFT	%LNT
**Antitrypsin**	**Coefficient**	0.09013	0.04837	0.12542	0.13905	-0.05011	0.01436	0.03648	-0.07845	-0.08442
	**p-value**	0.0227	0.2221	0.0015	0.0004	0.2058	0.7171	0.3573	0.0474	0.0329
**IgA**	**Coefficient**	0.06158	0.05146	0.16764	0.09986	-0.07978	0.06306	-0.06645	0.01217	-0.00117
	**p-value**	0.12	0.1939	<.0001	0.0115	0.0438	0.1113	0.0933	0.7589	0.9765
**Lactalbumin**	**Coefficient**	0.04537	-0.00662	0.12868	0.18484	0.01084	0.03771	0.01469	-0.16295	0.07825
	**p-value**	0.2522	0.8673	0.0011	<.0001	0.7844	0.3413	0.7108	<.0001	0.048
**Lactoferrin**	**Coefficient**	0.20304	0.08045	0.05055	0.06184	-0.07269	-0.03986	0.02572	-0.0255	-0.03425
	**p-value**	<.0001	0.0421	0.2019	0.1184	0.0663	0.3144	0.5163	0.5199	0.3873
**Lysozyme**	**Coefficient**	0.03992	-0.01906	0.10655	0.07536	-0.00403	-0.02997	-0.12118	-0.03826	0.04191
	**p-value**	0.3136	0.6305	0.007	0.0569	0.9191	0.4494	0.0022	0.3342	0.2902
**Osteopontin**	**Coefficient**	0.06398	-0.00183	0.05333	0.04086	0.00507	-0.09073	-0.00681	-0.18391	-0.02355
	**p-value**	0.1061	0.9631	0.1782	0.3024	0.8982	0.0218	0.8636	<.0001	0.5524

		%LNnT	%LNT + LNnT	%LNFP II	%LNFP I + III	%LNH	%LNnH	%p-LNH	%MFpLNH IV	%4120a	%MFLNH III + I
**Antitrypsin**	**Coefficient**	-0.06466	-0.10733	-0.07207	-0.04497	0.02334	0.03022	0.02444	-0.08557	-0.162	-0.08964
	**p-value**	0.1024	0.0066	0.0686	0.2564	0.556	0.4457	0.5374	0.0306	<.0001	0.0234
**IgA**	**Coefficient**	-0.12259	-0.04513	-0.00572	0.00106	-0.12993	-0.13935	-0.0852	-0.11612	-0.00433	-0.10387
	**p-value**	0.0019	0.2546	0.8853	0.9786	0.001	0.0004	0.0313	0.0033	0.9129	0.0086
**Lactalbumin**	**Coefficient**	-0.18791	-0.02639	-0.04737	-0.08873	0.05044	-0.05519	-0.03772	-0.14212	-0.20181	0.01505
	**p-value**	<.0001	0.5055	0.2318	0.0249	0.2029	0.1635	0.3411	0.0003	<.0001	0.7042
**Lactoferrin**	**Coefficient**	-0.13712	-0.10047	0.0186	0.10074	-0.03215	-0.06299	0.07442	-0.19905	-0.03502	-0.14209
	**p-value**	0.0005	0.011	0.6388	0.0108	0.4173	0.1117	0.0601	<.0001	0.3768	0.0003
**Lysozyme**	**Coefficient**	-0.07494	-0.01048	0.00372	-0.02628	0.04331	-0.06325	-0.11697	-0.01659	0.02644	0.05687
	**p-value**	0.0583	0.7914	0.9253	0.5073	0.2743	0.1102	0.0031	0.6756	0.5047	0.151
**Osteopontin**	**Coefficient**	-0.1041	-0.08093	-0.09178	-0.0286	0.12793	0.01881	0.07697	-0.0352	-0.10565	-0.01274
	**p-value**	0.0084	0.0409	0.0203	0.4704	0.0012	0.6352	0.0518	0.3743	0.0075	0.7478

		%IFLNH III	%IFLNH I	%DFpLNH II	%DFLNHb	%DFLNHa	%DFLNHc	%TFLNH	%4320a	%5130a	%5130b
**Antitrypsin**	**Coefficient**	-0.03713	-0.22323	-0.09591	-0.11146	0.02065	-0.22692	-0.0472	-0.21693	0.00561	-0.1489
	**p-value**	0.3487	<.0001	0.0153	0.0048	0.6024	<.0001	0.2335	<.0001	0.8874	0.0002
**IgA**	**Coefficient**	-0.13754	-0.05397	-0.03175	-0.04484	-0.04033	-0.0194	0.02558	0.0341	-0.07927	-0.08119
	**p-value**	0.0005	0.173	0.423	0.2577	0.3087	0.6245	0.5187	0.3895	0.0452	0.0402
**Lactalbumin**	**Coefficient**	-0.16842	-0.33905	-0.19342	-0.11599	-0.01243	-0.32062	-0.10934	-0.34059	0.11168	-0.23073
	**p-value**	<.0001	<.0001	<.0001	0.0033	0.7538	<.0001	0.0057	<.0001	0.0047	<.0001
**Lactoferrin**	**Coefficient**	-0.24473	-0.05492	-0.1466	-0.18269	0.06438	-0.04399	0.07516	0.00323	-0.03618	-0.11945
	**p-value**	<.0001	0.1655	0.0002	<.0001	0.104	0.2669	0.0576	0.935	0.3612	0.0025
**Lysozyme**	**Coefficient**	0.0139	-0.1224	0.01088	0.03257	-0.0256	-0.19867	0.00476	-0.18537	0.10016	-0.12389
	**p-value**	0.7258	0.0019	0.7837	0.4111	0.5182	<.0001	0.9044	<.0001	0.0113	0.0017
**Osteopontin**	**Coefficient**	-0.07979	-0.2081	-0.09771	-0.07439	0.03039	-0.23983	-0.08254	-0.19597	0.08332	-0.20225
	**p-value**	0.0438	<.0001	0.0135	0.0602	0.4431	<.0001	0.037	<.0001	0.0352	<.0001

		%F-LNO	%5130c	%DFLNO I	%DFLNnO II	%5230a	%DFLNnO I + DFLNO II	%5230 + DLFNO1	%5230b	%5330a	%4240a
**Antitrypsin**	**Coefficient**	-0.05805	-0.26511	0.00098	-0.07974	-0.0026	0.16716	0.14299	0.15104	0.32447	0.32427
	**p-value**	0.1427	<.0001	0.9802	0.0439	0.9477	<.0001	0.0003	0.0004	<.0001	<.0001
**IgA**	**Coefficient**	-0.14552	-0.00868	-0.11661	0.0421	0.11412	-0.12638	-0.02344	0.04811	0.03682	0.02186
	**p-value**	0.0002	0.8267	0.0032	0.2879	0.0039	0.0014	0.5542	0.2596	0.3527	0.5812
**Lactalbumin**	**Coefficient**	-0.15544	-0.32629	0.14807	-0.14192	-0.14305	0.17141	0.09864	0.09517	0.40756	0.38803
	**p-value**	<.0001	<.0001	0.0002	0.0003	0.0003	<.0001	0.0126	0.0255	<.0001	<.0001
**Lactoferrin**	**Coefficient**	-0.15718	-0.04974	-0.0945	0.03218	0.01227	0.11247	0.11041	-0.08201	0.23421	0.22715
	**p-value**	<.0001	0.2092	0.0169	0.4167	0.757	0.0044	0.0052	0.0544	<.0001	<.0001
**Lysozyme**	**Coefficient**	0.02578	-0.32133	0.05458	0.09599	0.34301	0.00325	0.24666	0.20771	0.00474	0.00358
	**p-value**	0.5154	<.0001	0.1682	0.0152	<.0001	0.9346	<.0001	<.0001	0.9047	0.928
**Osteopontin**	**Coefficient**	-0.07911	-0.31812	-0.04039	-0.06	0.13005	0.146	0.20733	-0.00187	0.33575	0.34673
	**p-value**	0.0456	<.0001	0.308	0.1298	0.001	0.0002	<.0001	0.9651	<.0001	<.0001

		%5300a	%6400a	%6400b	%6SL	%3SL	%LSTc	%LSTb	%LSTa	%F-LSTc	%S-LNH
**Antitrypsin**	**Coefficient**	0.09877	0.32239	0.14199	-0.08391	-0.07009	0.02717	-0.0121	0.00206	0.06551	-0.01549
	**p-value**	0.0125	<.0001	0.0003	0.034	0.0766	0.4929	0.7601	0.9586	0.098	0.6959
**IgA**	**Coefficient**	-0.15458	0.03714	-0.01069	0.12969	0.11168	0.10691	0.05258	-0.12589	-0.06405	0.00845
	**p-value**	<.0001	0.3486	0.7875	0.001	0.0047	0.0068	0.1844	0.0014	0.1058	0.8312
**Lactalbumin**	**Coefficient**	0.05102	0.37814	0.12655	-0.12918	-0.17315	-0.01887	0.04	0.07811	0.10875	0.03278
	**p-value**	0.1978	<.0001	0.0013	0.0011	<.0001	0.6339	0.3127	0.0484	0.0059	0.4081
**Lactoferrin**	**Coefficient**	-0.02964	0.22985	0.10855	0.10599	-0.17697	0.06299	-0.03365	-0.11254	0.00647	0.01584
	**p-value**	0.4546	<.0001	0.006	0.0073	<.0001	0.1116	0.3958	0.0044	0.8703	0.6893
**Lysozyme**	**Coefficient**	-0.06538	0.00578	-0.01657	-0.06583	0.1154	-0.06202	0.07621	0.00803	-0.20735	0.0015
	**p-value**	0.0987	0.8841	0.6759	0.0964	0.0035	0.1173	0.0542	0.8395	<.0001	0.9699
**Osteopontin**	**Coefficient**	0.10038	0.34548	0.13769	0.08988	-0.0603	-0.09394	-0.03541	0.06637	-0.05606	-0.00301
	**p-value**	0.0111	<.0001	0.0005	0.0231	0.1279	0.0175	0.3715	0.0937	0.1569	0.9394

		%4021+ S-LNnH II	%DFS-LNnH	%5311a	%4211a	%4211b	%4211c	%4100a	%4100b	Antitrypsin	IgA6
**Antitrypsin**	**Coefficient**	0.09493	0.18724	0.32071	0.18159	0.18239	-0.01732	0.01793	0.06556		0.08706
	**p-value**	0.0164	<.0001	<.0001	<.0001	<.0001	0.685	0.6745	0.1243		0.0278
**IgA**	**Coefficient**	-0.00851	-0.11254	0.01198	-0.12082	-0.11494	0.0965	-0.11473	0.09905	0.08706	
	**p-value**	0.83	0.0082	0.7625	0.0045	0.0069	0.0235	0.007	0.02	0.0278	
**Lactalbumin**	**Coefficient**	0.02244	0.3197	0.39559	0.32389	0.32215	-0.03271	0.01001	0.15569	0.34856	0.05303
	**p-value**	0.5712	<.0001	<.0001	<.0001	<.0001	0.4435	0.8146	0.0002	<.0001	0.1806
**Lactoferrin**	**Coefficient**	0.10624	0.12329	0.22313	0.11852	0.11742	-0.0785	-0.16708	0.05579	0.31261	0.50298
	**p-value**	0.0072	0.0037	<.0001	0.0053	0.0058	0.0656	<.0001	0.191	<.0001	<.0001
**Lysozyme**	**Coefficient**	-0.05718	0.00143	-0.00602	0.00294	0.00125	-0.02792	-0.05073	0.09188	0.24488	0.16822
	**p-value**	0.1488	0.9733	0.8793	0.9451	0.9767	0.513	0.2345	0.031	<.0001	<.0001
**Osteopontin**	**Coefficient**	0.06645	0.2662	0.34675	0.26555	0.26715	-0.1086	-0.09432	-0.05455	0.37555	0.2336
	**p-value**	0.0933	<.0001	<.0001	<.0001	<.0001	0.0107	0.0268	0.201	<.0001	<.0001

		Lactalbumin	Lactoferrin	Lysozyme	Osteopontin
Antitrypsin	Coefficient	0.34856	0.31261	0.24488	0.37555
	p-value	<.0001	<.0001	<.0001	<.0001
IgA	Coefficient	0.05303	0.50298	0.16822	0.2336
	p-value	0.1806	<.0001	<.0001	<.0001
Lactalbumin	Coefficient		0.16708	0.11339	0.29788
	p-value		<.0001	0.0041	<.0001
Lactoferrin	Coefficient	0.16708		0.30991	0.54752
	p-value	<.0001		<.0001	<.0001
Lysozyme	Coefficient	0.11339	0.30991		0.54986
	p-value	0.0041	<.0001		<.0001
Osteopontin	Coefficient	0.29788	0.54752	0.54986	
	p-value	<.0001	<.0001	<.0001	

Supplementary Table 2. Mean (SD) or prevalence (%) of Malawian infant morbidity and markers of inflammation at the 1st and 5th quintile of human milk oligosaccharide relative abundance for the exploratory analyses that were significant upon linear regression modeling. The tables are separated by secretor status. The tables including secretors are separated into tables containing morbidity and markers of inflammation. The tables including non-secretors are separated into tables containing incidence of morbidity, prevalence of morbidity, and markers of inflammation.

sample	HMO	Quintile	Incidence Illness 6-12 mo	Incidence Illness 6-7 mo	Incidence ARI 6-7 mo	Incidence Lost Appetite 6-12 mo	Prevalence Lost Appetite 6-12 mo	CRP 18mo	High CRP 6mo	High CRP 18mo
Full sample	%F-LNO	1	4.9 (2.9)	0.7 (0.8)	0.5 (0.7)					
Full sample	%F-LNO	5	5.9 (3.0)	1.0 (0.9)	0.7 (0.8)					
Full sample	%5230a	1		0.8 (1.0)					27.7	
Full sample	%5230a	5		1.1 (1.0)					36.1	
Full sample	%4211b	1				1.5 (1.7)	6.0 (7.6)			
Full sample	%4211b	5				1.3 (1.8)	4.5 (7.8)			
Full sample	%5130c	1		1.0 (0.9)					34.3	
Full sample	%5130c	5		0.7 (0.8)					24.7	
Full sample	%4211a	1		0.7 (0.8)	0.5 (0.6)					
Full sample	%4211a	5		0.9 (1.0)	0.7 (0.9)					
Full sample	%6400a	1						6.2 (11.9)		
Full sample	%6400a	5						16.0 (34.3)		
Full sample	%6400b	1								33.9
Full sample	%6400b	5								39.2

sample	HMO	Quintile	Fever 6-7 mo	Diarrhea 6-7 mo	Incidence ARI 6-7 mo	Incidence Illness 6-7 mo	Incidence Diarrhea 6-12 mo	Incidence ARI 6-12 mo	Incidence Illness 6-12 mo	Prevalence Diarrhea 6-12 mo	Prevalence ARI 6-12 mo	Prevalence Illness 6-12mo
Secretors	%LNFP I+III	1		15.9								
Secretors	%LNFP I+III	5		12.0								
Secretors	%LNFP II	1		11.8	0.7 (0.8)	0.9 (1.0)						
Secretors	%LNFP II	5		28.4	1.0 (0.9)	1.2 (1.0)						
Secretors	%3'SL	1	43.0				2.3 (2.9)			6.9 (8.4)		
Secretors	%3'SL	5	24.1				1.6 (2.2)			4.4 (7.3)		
Secretors	%LNDFH + 3120	1		11.6	0.6 (0.8)	0.8 (0.9)						
Secretors	%LNDFH + 3120	5		23.2	1.0 (0.9)	1.1 (0.9)						
Secretors	%DFpLNH II	1		10.0			1.6 (2.1)					
Secretors	%DFpLNH II	5		23.0			2.1 (3.1)					
Secretors	%LNH	1		26.2								
Secretors	%LNH	5		11.0								
Secretors	%TFLNH	1		11.8								
Secretors	%TFLNH	5		24.1								
Secretors	%5130c	1					2.0 (3.2)			5.8 (9.2)		
Secretors	%5130c	5					1.7 (2.6)			5.2 (8.6)		
Secretors	%F-LSTc	1		10.6								
Secretors	%F-LSTc	5		24.4								
Secretors	%DFLNHc	1				1.0 (0.9)	1.8 (2.4)		5.7 (3.1)			
Secretors	%DFLNHc	5				0.8 (0.8)	1.7 (2.7)		5.1 (2.9)			
Secretors	%5230b	1			1.0 (0.9)	1.3 (1.1)	2.6 (3.3)		6.4 (3.7)	7.7 (9.3)		
Secretors	%5230b	5			0.5 (0.7)	0.7 (0.8)	1.3 (1.8)		5.1 (2.6)	3.6 (5.2)		
Secretors	%S-LNH	1				1.1 (1.0)	2.0 (2.3)		6.1 (3.7)			
Secretors	%S-LNH	5				0.7 (0.8)	1.2 (1.9)		4.5 (2.7)			
Secretors	%4100b	1					1.9 (2.4)		5.8 (3.8)			28.5 (22.0)
Secretors	%4100b	5					1.6 (2.1)		5.3 (2.7)			23.8 (18.7)
Secretors	%4240a	1			0.9 (0.9)			4.7 (2.7)			25.9 (24.1)	31.5 (25.6)
Secretors	%4240a	5			0.6 (0.8)			3.8 (3.4)			17.4 (17.3)	23.9 (19.7)

sample	HMO	Quintile	CRP 6mo	AGP 6mo	AGP 18mo	High AGP 6mo	High AGP 18mo	High CRP 6mo	High CRP 18mo
Secretors	%LNFP II	1		1.2 (0.5)					
Secretors	%LNFP II	5		1.3 (0.4)					
Secretors	%3'SL	1				50.8			
Secretors	%3'SL	5				69.9			
Secretors	%LNDFH + 3120	1		1.2 (0.5)					
Secretors	%LNDFH + 3120	5		1.3 (0.5)					
Secretors	%S-LNH	1					80.5		
Secretors	%S-LNH	5					58.0		
Secretors	%3FL	1	7.4 (13.6)						
Secretors	%3FL	5	12.4 (34.6)						
Secretors	%DFLNO I	1						23.3	
Secretors	%DFLNO I	5						36.1	
Secretors	%LSTb	1						22.4	
Secretors	%LSTb	5						36.6	
Secretors	%5130a	1					71.8		
Secretors	%5130a	5					56.2		
Secretors	%5230a	1			1.4 (0.5)		67.1		
Secretors	%5230a	5			1.5 (0.6)		80.5		
Secretors	%6400a	1							32.1
Secretors	%6400a	5							45.0
Secretors	%2'FL	1						41.3	
Secretors	%2'FL	5						26.0	
Secretors	%5300a	1						27.3	
Secretors	%5300a	5						22.4	

Sample	HMO	Quintile	Fever 6-7 mo	Diarrhea 6-7 mo	Lost Appetite 6-7 mo	Incidence ARI 6-7 mo	Incidence Illness 6-7 mo	Incidence Diarrhea 6-12 mo	Incidence Lost Appetite 6-12 mo	Incidence ARI 6-12 mo	Incidence Fever 6-12 mo	Incidence Illness 6-12 mo
Non-secretors	%Undecorated HMOs	1		0.0	4.0							
Non-secretors	%Undecorated HMOs	5		20.0	16.0							
Non-secretors	%LNnT	1		3.6					0.7 (1.2)			
Non-secretors	%LNnT	5		32.0					1.5 (2.0)			
Non-secretors	%fuc+sial HMOs	1		16.7	25.0							
Non-secretors	%fuc+sial HMOs	5		0.0	7.4							
Non-secretors	%DFLNH_b	1		26.9	19.2		0.8 (0.7)					
Non-secretors	%DFLNH_b	5		3.8	7.7		0.5 (0.6)					
Non-secretors	%MFLNH III+I	1					0.8 (0.7)					
Non-secretors	%MFLNH III+I	5					0.4 (0.6)					
Non-secretors	%LSTb	1		25.9								
Non-secretors	%LSTb	5		0.0								
Non-secretors	%LNDFH + 3120	1			20.0							
Non-secretors	%LNDFH + 3120	5			11.1							
Non-secretors	%DFpLNH II	1									1.3 (1.5)	
Non-secretors	%DFpLNH II	5									2.6 (2.6)	
Non-secretors	%LNnH	1		0.0	6.7							
Non-secretors	%LNnH	5		20.0	28.0							
Non-secretors	%IFLNH III	1							0.6 (0.9)			
Non-secretors	%IFLNH III	5							1.8 (2.3)			
Non-secretors	%LNH	1			0.0							
Non-secretors	%LNH	5			23.1							
Non-secretors	%DFLNO I	1	47.8									
Non-secretors	%DFLNO I	5	28.6									
Non-secretors	%5130a	1	40.7								1.9 (1.8)	

Sample	HMO	Quintile	Fever 6-7 mo	Diarrhea 6-7 mo	Lost Appetite 6-7 mo	Incidence ARI 6-7 mo	Incidence Illness 6-7 mo	Incidence Diarrhea 6-12 mo	Incidence Lost Appetite 6-12 mo	Incidence ARI 6-12 mo	Incidence Fever 6-12 mo	Incidence Illness 6-12 mo
Non-secretors	%5130a	5	17.2								1.3 (1.4)	
Non-secretors	%3FL	1									1.2 (1.3)	
Non-secretors	%3FL	5									2.6 (2.2)	
Non-secretors	%TFLNH	1			22.2							
Non-secretors	%TFLNH	5			3.7							
Non-secretors	%p-LNH	1		3.8	15.4							
Non-secretors	%p-LNH	5		24.0	24.0							
Non-secretors	%5230a + DFLNnO I/DFLNO II	1									1.4 (1.4)	
Non-secretors	%5230a + DFLNnO I/DFLNO II	5									2.5 (2.0)	
Non-secretors	%F-LNO	1		3.6					0.8 (1.3)		1.4 (1.9)	
Non-secretors	%F-LNO	5		22.2					1.8 (2.2)		1.9 (1.6)	
Non-secretors	%4021a + S-LNnH II	1		3.6	10.7							
Non-secretors	%4021a + S-LNnH II	5		20.8	25.0							
Non-secretors	%5300a	1		0.0	10.0							
Non-secretors	%5300a	5		28.0	24.0							
Non-secretors	%6'SL	1						1.5 (1.8)	1.7 (1.8)		2.0 (1.8)	4.6 (2.4)
Non-secretors	%6'SL	5						1.2 (1.8)	0.4 (0.7)		1.5 (1.4)	3.8 (2.3)
Non-secretors	%5230a	1							0.9 (1.1)	2.4 (1.8)	1.6 (1.4)	3.9 (2.4)
Non-secretors	%5230a	5							2.1 (2.2)	4.2 (2.8)	2.8 (2.2)	5.4 (3.1)
Non-secretors	%S-LNH	1				0.7 (0.8)	1.0 (0.8)	1.8 (2.1)		3.2 (2.2)		4.7 (2.7)
Non-secretors	%S-LNH	5				0.3 (0.6)	0.5 (0.6)	0.8 (1.2)		3.3 (2.5)		4.4 (2.8)
Non-secretors	%5130c	1					0.8 (0.8)					
Non-secretors	%5130c	5					0.5 (0.7)					
Non-secretors	%5230b	1					0.9 (1.0)	1.3 (2.1)	0.7 (0.9)		1.4 (1.4)	
Non-secretors	%5230b	5					0.7 (0.9)	1.9 (1.8)	2.0 (2.0)		2.3 (2.5)	

Sample	HMO	Quintile	Fever 6-7 mo	Diarrhea 6-7 mo	Lost Appetite 6-7 mo	Incidence ARI 6-7 mo	Incidence Illness 6-7 mo	Incidence Diarrhea 6-12 mo	Incidence Lost Appetite 6-12 mo	Incidence ARI 6-12 mo	Incidence Fever 6-12 mo	Incidence Illness 6-12 mo
Non-secretors	%DFLNHa	1	41.7									
Non-secretors	%DFLNHa	5	29.6									
Non-secretors	%6400a	1							1.1 (1.4)		1.6 (1.5)	4.3 (2.6)
Non-secretors	%6400a	5							0.8 (1.5)		1.5 (1.4)	3.8 (3.5)
Non-secretors	%4211b	1							1.1 (1.2)		1.5 (1.5)	
Non-secretors	%4211b	5							1.0 (1.6)		1.6 (1.8)	
Non-secretors	%6400b	1							0.9 (1.1)			
Non-secretors	%6400b	5							0.9 (1.8)			
Non-secretors	%DFS-LNnH	1							1.1 (1.2)			
Non-secretors	%DFS-LNnH	5							1.0 (1.6)			
Non-secretors	%4240a	1							1.2 (1.4)			
Non-secretors	%4240a	5							0.8 (1.6)			

Sample	HMO	Quintile	Prevalence Illness 6-7 mo	Prevalence Diarrhea 6-12 mo	Prevalence Lost Appetite 6-12 mo	Prevalence ARI 6-12 mo	Prevalence Fever 6-12 mo	Prevalence Illness 6-12 mo
Non-secretors	%MFLNH III+I	1	5.7 (5.9)				6.7 (9.4)	
Non-secretors	%MFLNH III+I	5	3.2 (5.2)				4.5 (5.8)	
Non-secretors	%5130a	1					9.0 (10.9)	
Non-secretors	%5130a	5					4.4 (5.7)	
Non-secretors	%4100a	1				21.6 (15.4)		
Non-secretors	%4100a	5				14.4 (11.7)		
Non-secretors	%6'SL	1			6.6 (6.7)	16.2 (11.2)		
Non-secretors	%6'SL	5			1.3 (2.2)	13.7 (12.5)		
Non-secretors	%5230a	1			3.5 (4.8)	12.8 (10.5)		18.4 (12.6)
Non-secretors	%5230a	5			7.4 (7.3)	22.2 (17.1)		28.6 (20.7)
Non-secretors	%S-LNH	1		5.7 (6.9)	4.8 (6.5)	18.4 (16.2)		
Non-secretors	%S-LNH	5		2.1 (3.6)	2.6 (4.9)	16.7 (16.5)		
Non-secretors	%LDFT	1				17.6 (16.1)		
Non-secretors	%LDFT	5				17.4 (16.9)		
Non-secretors	%6400a	1			3.7 (5.9)		5.2 (5.1)	
Non-secretors	%6400a	5			2.9 (5.6)		5.5 (7.3)	
Non-secretors	%4211b	1			3.4 (4.1)		5.1 (5.1)	
Non-secretors	%4211b	5			3.7 (5.9)		5.6 (7.0)	
Non-secretors	%6400b	1			2.9 (3.8)			
Non-secretors	%6400b	5			3.2 (6.2)			
Non-secretors	%4240a	1			4.1 (6.0)			
Non-secretors	%4240a	5			2.8 (5.4)			

Sample	HMO	Quintile	CRP 6mo	CRP 18mo	AGP 6mo	AGP 18mo	High AGP 6 mo	High CRP 6 mo	High CRP 18 mo
Non-secretors	%α1-2-fucosylated HMOs	1		2.8 (3.9)	1.3 (0.4)		51.6		
Non-secretors	%α1-2-fucosylated HMOs	5		18.3 (30.6)	1.0 (0.4)		65.2		
Non-secretors	%Undecorated HMOs	1	7.3 (13.0)		1.1 (0.4)				
Non-secretors	%Undecorated HMOs	5	14.8 (27.1)		1.4 (0.5)				
Non-secretors	%fuc+sial HMOs	1						50.0	
Non-secretors	%fuc+sial HMOs	5						24.0	
Non-secretors	%DFLNH_b	1	15.0 (28.3)					42.9	
Non-secretors	%DFLNH_b	5	7.1 (13.0)					29.2	
Non-secretors	%LSTb	1		4.9 (11.5)		1.1 (0.5)			17.9
Non-secretors	%LSTb	5		13.8 (24.6)		1.5 (0.6)			42.9
Non-secretors	%LNDFH + 3120	1	14.6 (26.2)		1.5 (0.6)			48.1	
Non-secretors	%LNDFH + 3120	5	4.5 (11.2)		1.1 (0.4)			13.0	
Non-secretors	%3FL	1			1.4 (0.5)			44.8	
Non-secretors	%3FL	5			1.1 (0.4)			26.1	
Non-secretors	%TFLNH	1	12.6 (25.2)		1.3 (0.5)				
Non-secretors	%TFLNH	5	4.6 (9.6)		1.0 (0.3)				
Non-secretors	%S-LNH	1			1.0 (0.3)				
Non-secretors	%S-LNH	5			1.2 (0.4)				
Non-secretors	%DFS-LNnH	1							29.6
Non-secretors	%DFS-LNnH	5							41.7
Non-secretors	%LNT	1	7.0 (13.2)		1.1 (0.3)				
Non-secretors	%LNT	5	10.9 (24.5)		1.4 (0.5)				
Non-secretors	%LNFP II	1	14.0 (25.9)		1.4 (0.5)			50.0	
Non-secretors	%LNFP II	5	7.7 (16.9)		1.2 (0.4)			36.0	
Non-secretors	%LSTc	1	5.9 (11.3)						
Non-secretors	%LSTc	5	9.9 (24.1)						
Non-secretors	%F-LSTc	1		6.8 (11.2)	1.2 (0.4)	1.2 (0.4)			
Non-secretors	%F-LSTc	5		16.0 (28.9)	1.0 (0.3)	1.5 (0.7)			
Non-secretors	%DFLNnO II	1	10.8 (25.5)		1.3 (0.5)				
Non-secretors	%DFLNnO II	5	7.6 (17.4)		1.1 (0.3)				
Non-secretors	%5130b	1		14.0 (27.0)			69.2		
Non-secretors	%5130b	5		5.0 (10.7)			52.0		

Sample	HMO	Quintile	CRP 6mo	CRP 18mo	AGP 6mo	AGP 18mo	High AGP 6 mo	High CRP 6 mo	High CRP 18 mo
Non-secretors	%4211a	1							29.6
Non-secretors	%4211a	5							41.7

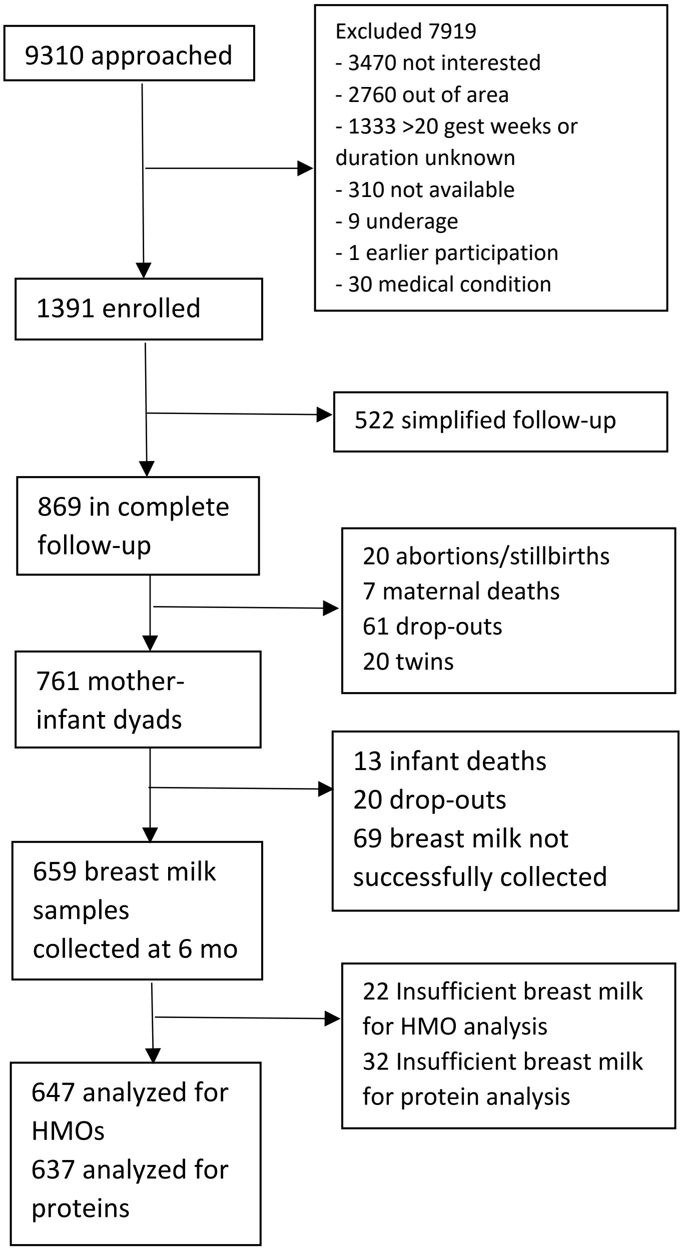

Supplementary Figure 1. Flow chart of participant enrollment and sample collection of Malawian women-infant dyads who participated in the International Lipid-Based Nutrient Supplement (iLiNS) Project and were included in the analysis of associations of Human Milk Oligosaccharides (HMOs) and bioactive breast milk proteins with infant morbidity and markers of inflammation.
